# The influence of miasmatic theory on the construction of the Hospital Geral de Santo António in Porto in the nineteenth century

**DOI:** 10.1590/S0104-59702024000100053

**Published:** 2024-10-14

**Authors:** Monique Palma

**Affiliations:** i Post-doctorate researcher, Centro Interuniversitário de História das Ciências e da Tecnologia/Nova School of Science and Technology. Caparica – Setúbal – Portugal; mpmoniquepalma@gmail.com

**Keywords:** Human agents, Non-human agents, Hospitals, Porto, Agentes humanos, Agentes não humanos, Transformações, Hospital, Porto

## Abstract

The aim of this work is to ascertain the relevance of the miasmatic theory for the construction and spatial organization of the Hospital Geral de Santo António in Porto, Portugal, in the 19th century, from then recent graduate physicians’ viewpoint. The methodology consists of analyzing the inaugural dissertations presented to the Escola Médico-Cirúrgica do Porto in order to assess the physicians’ perceptions of the Santo António Hospital building, with emphasis on the relationship between health, medicine and environment. This work intends to contribute to better understand the relationship between the theoretical referential approach to disease and medical practice, at the interface of health, medicine, and environment by analyzing interspecies encounters.

## Resumo

O objetivo deste trabalho é verificar a relevância da teoria miasmática para a construção e a organização espacial do Hospital Geral de Santo Antônio, no Porto, Portugal, no século XIX, sob o ponto de vista dos médicos recém-formados. A metodologia consistirá na análise das dissertações inaugurais apresentadas à Escola Médico-Cirúrgica do Porto, a fim de avaliar as percepções dos médicos sobre a construção do Hospital de Santo António, com ênfase na relação entre saúde, medicina e meio. O trabalho pretende contribuir para uma melhor compreensão da relação entre a abordagem teórica referencial da doença e a prática médica, na interface da saúde, da medicina e do ambiente ao analisar encontros interspécies.

Porto, August 19, 1799 – this marks the beginning of the operation of one of the most emblematic buildings (Lima, 1998) of the city. A place designed to restore health in a city that amid outbreaks of various diseases, such as tuberculosis and syphilis (Esteves, 2018; Couto, Esteves, 2018; Almeida, 2013), was undergoing transformation, experiencing commercial growth and demographic expansion (Silva, 2014). The occupation of the city and the occupation and performance of the hospital occurred through contact and deliberation – encounters – (Tsing, 2015), between different agents and conditions, both human and non-human, and circumstances of the environment, fostering interspecies collaboration, as commonly observed in the development of structures and dynamics that serve the human species (Tsing, 2015). This work is aligned with the theoretical and methodological approach of the History of Medicine within the perspective of the History of Science, Technology, and Medicine. This perspective emphasizes a history of interspecies relationship (Woods et al., 2018, p.11), recognizing that other animals and the environment are essential in the development of human history. They are integral to the study and daily practice of scientific and medical disciplines, influencing changes in socio-professional structure and technological innovation^1^ (Woods, et al., 2018; Tsing, 2015).

Between the 18th and 19th centuries, the human species gradually adjusted our ecosystem through the implementation of public health measures and the gradual adaptation of humans to various pathogen hosts (McNeill, 2000; Woods et al., 2018; Palma, Dias, Freitas, 2021). Hospitals and the miasmatic theory, which we will delve into later, were present and influenced physicians’ observations in making decisions on public policies during the period under analysis. This means that the environment and pathogens had an indirect presence in discussions on the implementation of public health policies and influenced the decisions made in the political and social spheres. This is the case with the choice of the location for the construction of the Hospital Geral Santo António [Santo António General Hospital] (HGSA), which we will discuss in another section of this article.

The primary sources used for this work are the Inaugural Dissertations, 53 of them, written between 1867 and 1899,^2^ and presented to the Escola Médico-Cirúrgica do Porto [Medical-Surgical School of Porto] (EMCP), founded in 1825. The newly graduated physicians^3^ who wrote these inaugural dissertations are not figures typically analyzed in conventional medical history – they are not agents of major discoveries and achievements. These physicians practiced medicine. They studied at the HGSA, where the EMCP was established in the city of Porto, and at the end of their studies, in order to obtain their medical diploma, they had to develop and defend a case study. Marginalized agents are rarely seen in Portuguese historiography (Palma, 2021a, 2021b; Abreu, 2021; Costa, Vieira, 2018; Vieira, 2016); however, quantitatively, they have a significant presence in the scope of the knowledge produced (Palma, 2021a).

In historiography, there is evidence of the perception of the HGSA building from the perspective of the physicians who used the hospital to study and practice their knowledge (Alves, Carneiro, 2007, p.27; Silva, 2014). This work aims to add to this topic of analysis, emphasizing marginalized agents in a research work that evokes the presence and understanding of non-human factors and agents (Palma, 2022; Palma, Dias, Freitas, 2021; Mason, 2018; Woods et al., 2018; Tsing, 2015; Lerner, Berg, 2015; Evans, Leighton, 2014), as actors in this case study. This will be our framework for inquiring about miasmatic theory and medical practice, at the interface of health, medicine, and the environment, for the construction and organization of the hospital space from the perspective of newly graduated physicians in Porto. To this end, we will first contextualize the miasmatic theory during the period under analysis, the context of the construction site of the HGSA, the architect responsible for the project and the alignment with medical premises, and then we will discuss the perception of newly graduated physicians at EMCP, whose academic headquarters were at the HGSA (Ferraz, 2013; Lemos, 1925; Monteiro, 1926).

## An air impregnated with miasmas

The importance attributed to the air is related to the miasmatic theory,^4^ which is rooted in the understanding of health in Hippocratic medicine (Benchimol, Santos, 2019, p.87; Amaral, 2018). Miasmas contained in putrid air were considered carriers of diseases (Palma, Dias, Freitas, 2021, p.238), hence the importance of pure air. In the modern perspective, the period during which the HGSA was designed and inaugurated, miasmas are seen as “environmental insults,” which Galen described as the six “non-naturals.”^5^ The first “non-natural” is corrupted air, the miasmas (Snowden, 2020, p.20).

As observed by historiography (Benchimol, Santos, 2019, p.87), when combining the miasmatic theory with the works of Antoine-Laurent de Lavoisier (1743-1794), who participated in the commission that studied the function of oxygen and carbon dioxide in the physiology of respiration, the basis for treating a diseased body involved demanding pure air, renewed air according to the characteristics of the environment and the body occupying that space, as stated by Jaime Larry Benchimol and Renata Soares C. Santos (2019) in “Hospitais, higiene e microbiologia no Rio de Janeiro: uma incursão histórica sob a ótica de Oswaldo Cruz” [Hospitals, hygiene, and microbiology in Rio de Janeiro: a historical incursion from the perspective of Oswaldo Cruz].^6^

It is recognized that both the miasmatic theory and the Hippocratic theory lost validation during the 19th century (Palma, 2022, 2021a; Snowden, 2020; Amaral, 2018; Pereira, Pita, 2006; Miguel, Reis, 2015; Porter, 1999), largely due to scientific advancements and experiments with non-human animals that allowed the consolidation of scientific medicine, which required these interspecies encounters and relations, as well as the presence and implementation of hygienic measures.^7^ It is worth remembering that, as indicated by Roy Porter (1999, p.303), great discoveries did not immediately revolutionize the daily medical-surgical practices in the modern period, such as William Harvey’s (1578-1657) discovery about blood circulation. While acknowledging the significance of these discoveries, Porter emphasized that their immediate impact on the daily exercise of healthcare agents might have been less radical (Palma, 2021a, p.21). It was in the 19th century that humanity witnessed the implementation and practical application of these discoveries in medical daily practice.

In addition to the fact that the 19th century was a transformative period, it was marked by the medical revolution launched by the École de Paris [School of Paris] (Snowden, 2020, p.204). Starting from the 1860s, the rise of bacteriology, especially associated with Louis Pasteur (1822-1895), in France, and Robert Koch (1843-1910), in Germany, established the role of pathogenic microorganisms (Porter, 1999, p.10-11). Nonetheless, the conceptual framework of the medical discipline was largely established by Hippocrates and Galen. Humoralism began to recede as physicians absorbed ideas about the circulatory and nervous systems, and the notion of contagion gained strength based on experiences with epidemic diseases (Snowden, 2020, p.204). However, the medical philosophy, therapeutics, and education still remained shaped in classical molds, with physicians and the population understanding epidemics through the doctrine of miasma “corruption” or poisoning of the air (p.204).

Within the perspective of the miasmatic theory, elements related to the environment, such as pure air and clean water, were considered important for restoring health, as evident in the inaugural dissertations analyzed, which form the documentary core of this work (Torres, 1884, p.42; Palma, 2023). Maria Antónia Pires de Almeida (2013), in her *Saúde pública e higiene na imprensa diária em anos de epidemias (1854-1918)* [Public health and hygiene in daily newspapers during epidemic years (1854-1918)], also found indications of the miasmatic theory in the newspapers she analyzed. According to Almeida (2013, p.52), the press contributed to disseminating hygiene practices, including the importance of keeping the house clean and well ventilated to eliminate “putrid miasmas,” which were considered the source of disease dissemination. The transition and coexistence of different medical theories was also occurring in other European countries (Snowden, 2020, p.257), such as in the case of Max von Pettenkofer (1818-1901), who, during the height of the cholera epidemic in Naples in 1884, relied on the miasmatic theory. For Pettenkofer, the danger that *Vibrio Cholerae* posed to Naples was not the contamination of the city’s drinking water, as suggested by John Snow (1813-1858) and Robert Koch (Snowden, 2020, p.257). In Pettenkofer’s system, the disease did not begin when the *Vibrios* entered the population’s intestines, but when they reached the groundwater beneath the city. There, under suitable conditions of temperature and humidity, they fermented and emitted poisonous fumes that the population inhaled (Snowden, 2020, p.257).

The miasmatic theory, which was part of medical understanding, declined during the 19th century, a trend observed not only in Portugal but also in other European countries. The 19th century was a period of recognized transition, with new advancements and the incorporation of discoveries that took place during the modern era. The space of the HGSA provided the physicians under analysis in this work with conditions for understanding medical practice and for questioning the prevailing medical theories. It is a representative place of the transitional period in medical thought that occurred in the European context.

## The location of the Hospital Geral de Santo António

The HGSA, the new hospital, was founded to meet the needs of a growing city and to replace the Royal Hospital, also known as Dom Lopo Hospital, which consisted of two wards (one with 42 beds for women and the other with 38 beds for men), as well as spaces for celebrating masses, a cloister, and a water fountain that served to supply the hospital^8^(Silva, 2014, p.714; Carr, 1769). Porto was constantly transforming and growing due to its commercial relations of Port wine and codfish, as well as small industries scattered throughout the city (Silva, 2014, p.710). Additionally, the city faced epidemics and diseases (Esteves, 2018; Couto, Esteves, 2018) that circulated among people and other animals, which further underscored the need for a larger hospital.

The work published by Helena Silva (2014), *O Porto e a construção da cidade moderna: o caso do Hospital Geral de Santo António, nos séculos XVIII e XIX* [Porto and the construction of the modern city: the case of Hospital General de Santo António in the eighteenth and nineteenth centuries], discusses the choice of the location for the construction of the HGSA. In this work, we can verify as well as consult the official document in the Santa Casa da Misericórdia [Holy House of Mercy]^9^Archive (Arquivo da Santa Casa de Misericórdia, ASCM) of Porto: royal letters issued by King José I (1714-1777) in the mid-18th century. By analyzing the royal letters issued by King José I, we find information about the choice of the location for the HGSA. The first letter we note was addressed to the Ombudsman, Officers and other members of the Board of the House of Mercy, which administered and was responsible for the HGSA (Silva, 2014; Esteves, 2018; Alves, Carneiro, 2007). The content of the letter established on June 12, 1767: “The relocation of said Hospital to the site at São Lazaro, which, being outside the walls and on higher ground, enjoys more benign and healthier airs”^10^(D. José, 1767).

On June 3, 1768, King José I sent another letter to the attention of the Santa Casa da Misericórdia, this time to indicate that the site at São Lázaro could not be the place for the construction of the hospital “because it was not deemed suitable for the intended building, as further inquiry showed regarding the waters for its foundation” (D. José, 1768). He authorized the construction to be made “on the land that includes the Rural Property,” stating that “it is situated outside the walls of that City between the fields of Cordoaria and Quarteis, since it has favorable circumstances of pure and healthy air” (D. José, 1768). Once again, an environmental factor, influenced the creation of urban infrastructure. Architecture had to respond to the needs and principles of health. This was a reality that occurred in several countries, including Brazil, Italy, Spain, England, France (Farah, Martin, 2023; Carnero Dorado, 2021; Benchimol, Santos, 2019, p.88). This change in location highlights the decision to comply with the principles advocated by the hygienist current for the construction of the HGSA (Silva, 2014, p.715). This allows us to observe the strong influence of non-human agents in political decision-making, such as in the case of choosing the site for the construction of the HGSA.

The construction of the HGSA meant a significant monetary investment from the Santa Casa da Misericórdia, which relied on donations to carry out the works. It is worth noting that part of the materials needed for the construction, essential in the 18th-century construction universe, was extracted from the environment, such as wood. For example, on January 19, 1777, João de Almada, as Ombudsman of the Santa Casa da Misericórdia, wrote and emphasized that the Hospital, being a work of interest to the Republic, needed wood to advance with the construction. He appealed to charitable help with donations of wood, whether oak, chestnut, or any other type of wood (Almada, 19 Jan. 1773). The mention of the Republic’s interest in the appeal for wood donations highlights the connection between human endeavors and the environment in the daily political activities – an interspecies encounter in the development of solutions for human society, emphasizing the reminder that we are interconnected with our surroundings (Woods et al., 2018; Tsing, 2015).

The architect responsible for the construction of the HGSA was John Carr of York (1723-1807). His selection may have been due to the good relationship of João de Almada e Melo (1703-1786) with England (Silva, 2014; Taylor, 1960; Ribeiro, 2012). Carr was recognized as an architect with experience in hospital architecture, as evidenced in a letter he wrote on November 5, 1769, to the Most Illustrious and Most Excellent D. António de Lencastre, where he stated: “I have designed many hospitals and more magnificent buildings than anyone else in England” (Carr, 5 Nov. 1769). Carr designed a Baroque building and introduced the neoclassical style in the city of Porto (Silva, 2014; Alves, Carneiro, 2007; Esteves, 2018), and the hospital continues to be in operation to this day.^11^

## Medical practice at the Hospital Geral de Santo António

The medical records we analyzed, consisting of inaugural dissertations (Palma, 2023),^12^ presented between 1867 and 1899, form the primary documentary source for this case study. These inaugural dissertations, defended and presented at the Escola Médico-Cirúrgica do Porto, constitute a vast and rich source for studies in the history of medicine (Palma, 2022; Costa, Vieira, 2018; Vieira, 2016). In the overall analysis, it was found that approximately 68% of the dissertations do not directly mention the hospital’s architecture as an element influencing medical practice or being unsuitable for medical treatment. The remaining approximately 32% address the topic proposed in this article, i.e., the challenges of medical practice in precarious hospital conditions and how the hospital environment facilitated the proliferation of miasmas. It is not the intention of this study to assert that the approximately 68% do not mention miasmas at all. Rather, the aim is to understand the direct relationship between the infrastructure of the HGSA and the proliferation of miasmas from the perspective of the newly graduated physicians who wrote the dissertations. The indication that approximately 32% of the cases address the situation of the hospital from the miasmatic perspective allows us to perceive how it corresponds to the decline that this theory experienced during the 19th century.

This percentage includes statements such as that made by Manoel Matos e Silva (1870) in his dissertation *Breves considerações sobre a podridão do hospital ou typho traumático* [Brief considerations on hospital rot or traumatic typhoid], that if the miasmatic infection of the air is not constantly the cause of rot, at least it always gives it the most dangerous character; these facts finally make known the influence of the individual’s state of health on the spontaneous march of this affection (p.27). Likewise, José Dias de Almeida Junior (1877, p.46), who argued about a current in the atmospheric layers, which would drag the miasma exhaled from the wards, and purify the environment, in his work *Hospitaes: necessidade d’um hospital barraca para a prática d’operações* [Hospitals: need for a tent hospital for surgical procedures].

These are examples of the type of information that was collected and which corresponds to the 32% we mentioned in the previous lines, as we can see in Graphic 1.

**Graphic 1:**
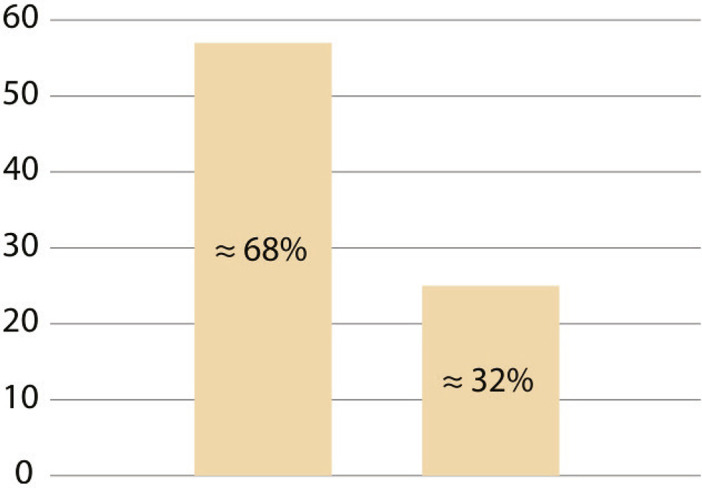
: Hospital infrastructure indication

Source: Palma (2023).

To understand these percentages, we need to contextualize part of the universe of sources at the time of their reproduction. For this purpose, we highlight: (a) the diversity of themes in the studies of newly graduated physicians from the EMCP; (b) a period in effervescence – indicative of the medical and sociopolitical context in Portugal; and (c) important elements for health and hospital construction – learning for the improvement of all.

### The diversity of themes in the studies of newly graduated physicians from the EMCP

The object of study in the inaugural dissertations analyzed here is diverse, as indicated by the titles of some of them, of which we present as examples: *A vacina animal como meio prophylactico therapeutico* [Animal vaccine as a prophylactic and therapeutic means](Castro, 1875); *O micróbio* [The microbe] (Cardoso, 1883); *A tuberculose considerada sob o ponto de vista da contagiosidade e parasitismo* [Tuberculosis considered from the perspective of contagion and and parasitism] (Freitas, 1887). From the aforementioned titles, it is possible to perceive the relationship between the medicine practiced at the HGSA and the indication and presence of the environment and other animals. This reinforces the framework of this analysis (Woods et al., 2018; Tsing, 2015), which emphasizes that human development is connected to other organisms and it is not solely a history of the human species.

The One Health initiative, for example, which understands that human health is related to the health of the environment and other animals, faces the challenge of having integrated data on human, animal, and environmental health. It is worth noting that the One Health approach predates the creation of the concept (Woods et al., 2018, p.15; Lerner, Berg, 2015). Here, we provide the necessary context, as the relationship between humans and other animals has changed immensely in recent years, but the interaction, advantages, and disadvantages of this coexistence are not new in history. Currently, there is a historiographic trend that seeks to analyze and better understand the presence of this type of information in medicine from periods prior to the present time (Woods et al., 2018), as is the case in this article, which focuses on an example from the 19th century. This allows us to contribute to the development of a historical outline of the One Health perspective, understanding that one of the challenges in this regard lies precisely in the fact that this initiative has multiple interpretations^13^ (Evans, Leighton, 2014) – which is also an asset due to the interdisciplinary nature of One Health.

The clarification we provide demonstrates that Portuguese physicians were aligned with the other components of the human ecosystem and how these other factors are strongly related to human health, and, consequently, to decision-making in health policies. Another title of an inaugural dissertation that we highlight, to conclude this point about the diversity of themes in the studies of newly graduated physicians from the EMCP, is authored by José Victorino de Sousa Albuquerque (1867), who, as indicated by historiography (Alves, Carneiro, 2007, p.27), presented the dissertation entitled *Condições hygienicas do hospital de Santo António do Porto com relação ás operações da grande cirurgia* [Hygienic conditions of the Hospital de Santo António do Porto regarding major surgical procedures], a study entirely focused on pointing out the conditions of hospital infrastructure, the environment, and its interference in the management of medical procedures.

### A period in effervescence: indicative of the medical and sociopolitical context in Portugal

Regarding the transformations that occurred during this period, we emphasize the context of medicine and the Portuguese sociopolitical context to gain insight into the universe of physicians who wrote the sources analyzed here. We agree with Cristiana Bastos’ assertion that nothing within the realm of health, suffering, and survival can be understood independently of its political and social inscription (Bastos, 2011).

In the case of medicine, physicians were required to write a dissertation at the end of their course, as stipulated in Article 154 of the Decree of April 23, 1840 (D. Maria II, 1840, p.119), under the rule of the second reign of Queen Maria II (1826-1827; 1834-1853), which aimed at regulating the medical-surgical schools of Lisbon and Porto. This requirement was not well received by medical students at the time, and it is common to find in the prefaces of their works an indication that the dissertation was simply fulfilling an obligation (Silva, 1870, p.17-18). Nevertheless, they left a legacy for the history of medicine from a period of recognized change in medical thinking and practice.

In the sociopolitical context, it is worth remembering that Portugal underwent a vast and complex process of transformation of its empire during the 19th century (Antunes, Polónia, 2016; Bastos, 2011). The country experienced the independence of one of its largest colonies, Brazil (Benchimol, Santos, 2019; Bastos, 2011). Additionally, parts of its continental and metropolitan territory were invaded by the French due to the campaigns (1807/1809/1810) of Napoleon Bonaparte (1769-1821), which led to the presence of British troops in Portuguese territory to assist the Portuguese forces (Bastos, 2011). The empire, which relied heavily on slavery, exploitation, and plantation, had to deal with various tumults and discontent, leading to a period of crisis that affected the Portuguese society (Bastos, 2011).

Amidst this turmoil, medicine, its agents, and actors coexisted. Portuguese medicine underwent organizational transformations since the mid-18th century (Palma, 2021a, 2021b; Abreu, 2021), such as the recognition of surgery alongside medicine, which was consolidated in practice during the 19th century. Today, it is acknowledged that surgery is the field of medicine that has driven innovation. Surgery serves as the starting point for the pursuit of mastery over health and disease (Porter, 1999). With the advent of microbiology, the miasmatic theory became increasingly relativized (Palma, 2022), significantly transforming medical thinking and practice (Porter, 1999).

These transformations were felt in the Portuguese context, as evidenced by the works of Ricardo Jorge (1858-1939) in the city of Porto and throughout Portugal (Amaral, 2018; Alves, 2008). Other non-human agents, invisible to our species’ eyes, began to be part of our understanding and contributed to the restructuring of medical thinking and practice. With this in mind, we can gain insight into some of the circumstances that are part of the overall analysis of the sources, which led us to find that approximately 68% of the dissertations do not make direct mention to miasmas and hospital architecture as elements interfering with medical practice or as being unsuitable for medical treatment due to their unhealthiness. The remaining approximately 32% provide indications about the difficulties of practicing medicine in precarious hospital conditions, where the physical structure of the hospital favored the presence of putrid air, which was believed to be the cause of diseases, the miasmas.

### Important elements for health and hospital construction: learning for the improvement of all

The hospital’s salubrity was meant to ensure that miasmas did not cause diseases (Silva, 1870, p.20). During that period, the fear of an unsanitary hospital also involved the dread of contagion through “Hospital Rot,” a gangrene that corrodes and consumes tissues, as its etymology indicates, annihilating their vitality (Silva, 1870, p.19).

In his dissertation titled *A phtisica, a Serra da Estrela e o específico do Dr. Kock* [The phthisic: the Serra da Estrela and the specific of Dr. Kock], José Alberto dos Santos Pimenta (1890, p.77) also pointed out the problem of material decomposition concentration observed in the hospital environment. In the city of Porto, he mentioned the existence of *ilhas* (islands), “nurseries where the human species lived in close proximity with various other animal species” (p.39). Pimenta discussed the experiences carried out on other animals and the need to take care when coexisting with different species in the urban environment of Porto – he brought in these examples as part of his argumentation in his dissertation to discuss medical technique innovations, insalubrity in confined spaces, and energy production.

Indeed, the interaction and contribution between different elements played a significant role in understanding various moments in the history of human medicine. The value of history lies in the learning and the opportunity to apply that knowledge for the improvement of all – humans and non-humans alike. This argument motivated the exploration of non-human agents in the history of medicine in this article, as it was observed that other physicians in different countries also made observations about non-human agents in the 19th century, namely Rudolf Virchow, in Germany, and William Osler, in Canada (Evans, Leighton, 2014, p.415).

### The circulation of air

The architect John Carr proved to be attentive and willing to design a hospital that adhered to the medical principles concerning the ideal circulation of air within a space, which, in this case, was associated with the miasmatic theory. To summarize, as already indicated by historiography (Silva, 2014; Alves, Carneiro, 2007), the architect designed a quadrangular hospital, with sides measuring 178 meters (north and south) and 172 meters (east and west), with a church at its center. Carr followed what was being done across Europe, placing Portugal on a par with the rest of the continent in terms of hospital infrastructure during the period under analysis. As noted by Helena Silva (2014, p.716), the design of the HGSA followed the model of hospitals in the United Kingdom, such as Guy’s Hospital in London (1722-1725), the Royal Naval Hospital in Haslar (1746-1761), and the Royal Naval Hospital in Plymouth (1758-1762). In the *Descrição da planta desenhada por John Carr para o Hospital do Porto* [Description of the plan designed by John Carr for the Hospital of Porto], the architect stated, for example, that: “Above all the infirmaries there shall be inserted ventilators and tubes from the ceiling through the roof to the open air; in order to obtain a perpetual current of air and evacuate the corrupted air from the infirmaries” (Carr, 1769). However, despite making such observations, in the analysis of the sources studied here, it is evident that, for the physicians, the idea of the building emphasized aesthetic architectural design. The assertion is made that: “The description of the planned construction of the Santo António Hospital, … shows that only one idea presided over such an endeavor – that of grandeur – paying little attention to the most important and necessary part, the conditions of hygiene and salubrity that an establishment of this order should have” (Albuquerque, 1867, p.21).

The description provided by José Victorino de Sousa Albuquerque, a newly graduated physician in 1867, can be consulted in the Santa Casa da Misericórdia’s Archive of Porto (Carr, 1769). In his dissertation, we can glimpse an internal hospital routine where the need for restructuring and urgent reforms in the HGSA building was evident from the perspective of the physicians:

On the right side of the corridor, there is first the kitchen, of which we will talk about in another article, and then there is the infirmary of St. Peter, reserved solely for syphilitic diseases in men.

We cannot, even with great effort, describe the impressions we felt when studying this infirmary!!! It is horrifying to see more than two dozen wretched individuals with emaciated faces, crowded in a small space, breathing air saturated with miasmas, with little light and no ventilation at all, struggling with diseases whose outcomes are so disastrous when sound hygiene and rigorous therapeutics does not provide valuable assistance (Albuquerque, 1867, p.36).

Albuquerque dedicated all the chapters of his inaugural dissertation to argue about the problems and challenges related to the environment and contact with pathogens within the HGSA building.

He made in his dissertation a detailed report specifying the size and capacity of the hospital interior, as we can see in Table 1, with “the indication of the average capacity of each window, the volume of each bed, the volume of the bench, and the total capacity, which includes the portion of air that the windows contain and the subtraction of air displaced by the beds and benches” (Albuquerque, 1867, p.43). The recognition of the need for fresh air was a certainty in 19th century hospital medicine. Figures like Peter Chalmers Mitchell, in London, and Carl Hagenbeck, in Hamburg, are known for improving ventilation and hygiene in hospitals in England and Germany because it was a crucial measure for health (Woods et al., 2018, p.36).

**Figure 2:**
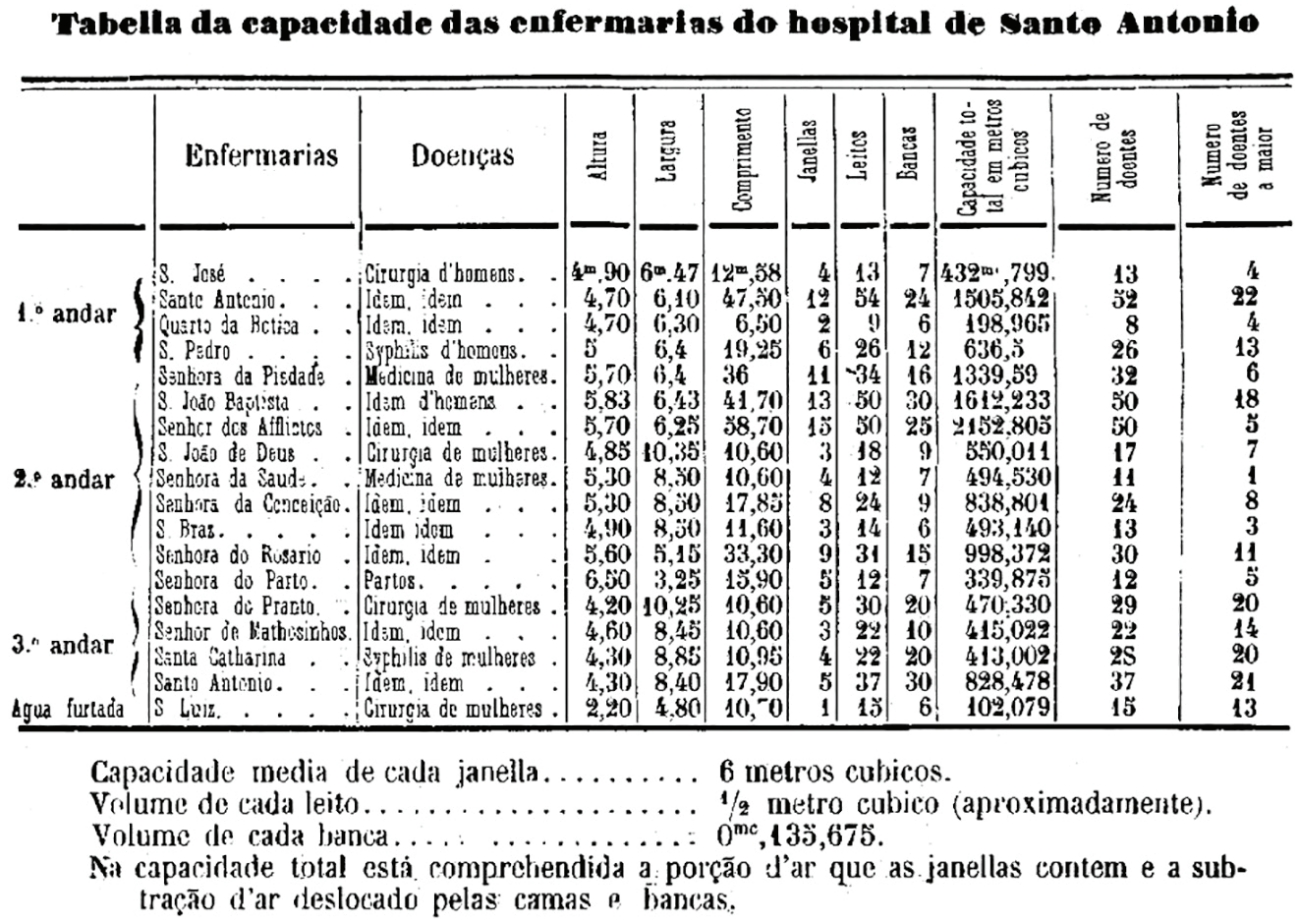
: Capacity of the infirmaries of the Santo António hospital (Albuquerque, 1867, p.43)

In England, in 1840, the concern with fresh air extended to the reform conducted at the London Zoo, with the premise of “preserving the animal in health” (Woods et al., 2018, p.36). Also in England, John Aikin (1747-1822), a surgeon, addressed the need to design hospital buildings where air was a matter of concern, and he advocated for the consideration of isolation wings and divisions for different diseases and patients (Silva, 2014, p.720). Some physicians in the sources analyzed in this study commented on John Aikin’s ideas in an attempt to understand or assist in finding the best solution for the HGSA (Almeida Júnior, 1877, p.52; Albuquerque, 1867, p.25). By finding this kind of reference, it is evident that the physicians from the Escola Médico-Cirúrgica do Porto were aligned with developments in other countries and medical knowledge being published elsewhere, particularly in Europe.

There were surveys to issue an opinion on the situation of the hospital, as seen in works such as *O Hospital da Santa Casa da Misericórdia do Porto ou proposta apresentada em mesa no dia 2 de Janeiro de 1865 por João Mendes Osório, mesário que então era da mesma Santa Casa e a contra-proposta apresentada ulteriormente pelo Mordomo das Obras* [The Santa Casa da Misericórdia do Porto Hospital or the proposal presented at the board on January 2, 1865, by João Mendes Osório, Chairman of the Board who was then a member of the institution and the counter-proposal presented later by the Steward of the works] (Osório, 1868). And, approximately two decades later, *O Hospital de Santo António da Misericórdia do Porto: relatório* [The Santa Casa da Misericórdia do Porto Hospital: Report], by António Augusto da Costa Simões (1883). Both works provide careful observations about the need for restructuring the HGSA (Silva, 2014; Couto, Esteves, 2018). Even in the context of the second half of the 19th century, mentions and concerns about miasma are evident. Mendes Osório (1868, p.XXVI-XXVII/7) stated that “miasmas resulting from the decomposition of organic debris brought by currents and cryptogamic formations created on humid surfaces of the walls were being dragged in.” Costa Simões (1883, p.286) also did not shy away from the miasmatic thinking and proposed another ventilation system to prevent the accumulation of miasmas in the hospital wards. His references included French authors such as Jules Félix, Frédéric Jaeger (1838-1878), Émile Sabouraud (1837-1892) – *Étude sur les hôpitaux-baraques*, 1872 –, and Amédée Chassagne – *Les hopitaux sans étages et à pavillons isolés*, 1878.

It is interesting to note that the process of knowledge circulation between Portugal and France does not seem to have suffered penalties or interruptions, even during the French invasion of Portuguese territory. The references to developments in France are strongly present in the sources discussed in this work. However, our focus is to understand how, even when using a theory that lost its strength in medicine in the 19th century, it was still used in arguments that called for structural reforms in the HGSA, which shows how the physical space and its environmental conditions were significant elements in the medical argumentation, leading to the demand for the renewal of health policies.

### The marshy area and the proximity to the river

Despite the twists and turns in King José I’s decision about the hospital’s location, the analysis of the sources suggests that the king did not receive the best advice at the time. This is because the chosen location for the hospital was also considered unsuitable “because, as we all know, it was built on marshy ground, which is the main reason why the construction is so far behind schedule” (Osório, 1868, p.XXXVI-XXXVII/7). The statement that the hospital was built on marshy ground was also made by physicians who wrote the inaugural dissertations from the documentary universe we analyzed (Almeida Júnior, 1877, p.46; Silva, 1870, p.26). Albuquerque (1867) questioned whether the fact that the hospital had been built on a swamp was one of the main causes of insalubrity. According to him, in 1867, there were doubts “that the swamp still existed due to the solidity of the walls of such a large building, the nature of the soil and subsoil that prevail in the rest of this neighborhood and even throughout the city, leading us to believe that the swamp is completely drained” (p.28). Draining swamps was a public health measure in Portugal in the 19th century (Palma, Dias, Freitas, 2021). In England, drained land was used for the construction of buildings other than hospitals (Woods et al., 2018). While it was not possible to verify the accuracy of this claim, it is evident that it was a factor that raised concern among Portuguese physicians, as they believed that the humidity emanating from the ground was a strong enough element to corrupt the air inside the hospital (Almeida Júnior, 1877, p.47).

The approach of José Dias de Almeida Júnior in 1877 is also noteworthy. He argued that the ideal solution for the city of Porto would be the creation of a “hospital hut” for surgical practice (Almeida Júnior, 1877). The young physician apologized to those well-meaning individuals involved in the construction of the HGSA, as what they wished the project to be was not reflected in reality. Almeida Júnior (1877, p.46) commented on the advantage or disadvantage of the distance between the hospital and the river, stating that it was of little importance “to know that a stream of water, crossing such a building, could produce a current in the atmospheric layers that would carry away the miasmas exhaled from the wards and purify the environment where the patients were located.” The physician argued that expecting to find health in the HGSA could be considered an act of audacity (p.35), given the lack of conditions at the site. Nevertheless, the newly graduated physician, in addition to his negative criticism, used his graduation case study to propose a possible solution to the humidity problems, arguing that “vegetation could modify the soil’s humidity, purify the atmosphere, and partially mitigate the identified issues, as stated by Chevreul in Annales d’Hygiène: ‘Si l’utilité des arbres, pour prévenir la denudation des terrains en pente, atténuer les effets des pluies d’orage ou des pluies nuisibles par leur continuité …’” (p.47). It is interesting to note that vegetation in humid areas served both to address health-related issues and environmental concerns in the 19th century, as was the case with foresting dunes for sand fixation (Palma, Dias, Freitas, 2021), as medicine and environment were interrelated to solve different circumstances.

## Final considerations

This article serves as an addition to the group of analyses concerning the perspective of physicians on the space of the Hospital Geral de Santo António. It is not possible, nor is it intended, to exhaustively cover a topic in a single case study. Our goal was to gain insight within the framework of the History of Science, Technology, and Medicine which values the interaction between human and non-human agents (Palma, 2022; Palma, Dias, Freitas, 2021; Mason, 2018; Woods et al., 2018; Tsing, 2015; Lerner, Berg, 2015; Evans, Leighton, 2014), to examine the observations of newly graduated physicians, a group often overlooked by conventional historiography. The requirement for the submission of an inaugural dissertation imposed by the decree of Queen Maria I allows us to bring these men out of anonymity. It is worth noting that the term “men” is used here because there were no dissertations authored by women in the group of source documents analyzed and presented in this article.

When examining the miasmatic theory and its impact on medical practice, at the interface of health, medicine, and environment for the construction and organization of space from the perspective of physicians, we can observe a period of great transformations in the 19th century. The city of Porto experienced demographic and industrial growth (Esteves, 2018; Silva, 2014), which also led to increased contact and adaptation of the human species to other non-human agents, resulting in the contamination and spread of diseases. The environment and other animals were present in the universe of sources analyzed in this study. According to the newly graduated physicians, human health was interconnected with other elements of the ecosystem. The encounter with other species and the environment allowed for the formulation of medical thoughts and brought attention to the structural problems in the functioning of the Hospital Geral de Santo António. As we hope to have demonstrated, the miasmatic theory served as a direct criticism of the hospital building. The foul and miasmatic airs needed to be eradicated because the physicians believed they were linked to the spread of diseases.

The focus of this article was to examine the case of the HGSA and analyze the influence of miasmas from a human health-animal health-environment perspective as an inseparable group of interconnected relations, which have this connection bias present in medical works produced in the 19th century. We intend to give rise to reflection that allows us to equate that part of the value of history is in learning and in the opportunity to apply learning for the improvement of all (Evans, Leighton, 2014, p.417).

## NOTES

1 Bruno Latour developed the Actor-Network Theory (ANT) concept, which implies different processes to analyze interspecies co-relations. Bruno Latour created a procedure to analyze sources and the agents included in those sources. We respect and acknowledge Latour’s work with regard to the introduction of the topic of non-human agents, but we do not follow his type of ANT analysis. For more on ANT, see: Latour (1988, 2005).

2 A selection of dissertations was made, but it cannot be fully presented here due to limited space. Nevertheless, the list can be accessed in full in the database created in Zotero. See Palma (2023). Any difficulty in accessing it, please do not hesitate to contact us via e-mail.

3 It is of interest to collect and analyze the socio-professional profile of these agents, but the data collection and analysis are still ongoing. However, it is not the objective of the current research presented here.

4 The miasmatic theory is a widely explored subject in historiography. In this study, a synthesis of its definition is provided to contextualize our research object. Besides the works mentioned throughout the text, we recommend: Wood (1961); Ayliffe, English (2003); Cipolla (1992).

5 The second non-natural element was movement, or what today would be called exercise (or lack thereof). The other non-natural elements were sleep (or wakefulness), excretion (or retention), food and drink, and the passions of the soul (Snowden, 2020, p.20).

6 We do not have space to delve into hygienism in Portugal, but there are already many high-quality works developed on this topic, namely: Cosme (2006); Alves (2008).

7 For a detailed analysis, please refer to Silva (2014).

8 Santa Casa da Misericórdia, played an important role in the dynamics of healthcare in Portugal during the period under analysis in this paper. The administration of the hospitals was concentrated in Santa Casa da Misericórdia. For more information, see Amorim (2018).

9 The HGSA can be visited virtually. A work developed within the project funded by the Directorate-general for Cultural Heritage: ProMuseus. See Museum of the Hospital Center of Porto (5 Mar. 2020).

10 In this and other citations from Portuguese, a free translation has been provided.

11 As mentioned in a previous note, the 83 sources analyzed for this study can be consulted. See Palma (2023) Any difficulty in accessing it, please do not hesitate to contact us via e-mail.

12 Evan and Leighton (2014), in an article titled “A history of One Health,” discuss the interconnection and convergence that exist and consider various factors for understanding One Health – the concept that human health is interconnected with animal and environmental health. This approach involves factors such as epidemiological globalization, the adaptation of pathogens, food insecurity, changes in human demography, evolution of animal production systems, and climate change, among others. The authors reflect on the complexity and the need to better understand the relationship between humans, animals, and the environment.

13 The concept of One Health has been implemented in the daily healthcare practice in Portugal through the Portuguese National Health Service (SNS, 2023). The SNS portal published a note titled “São João pioneers the approach to the concept of animal, environmental, and human health as One Health with integrated solutions,” in which the One Health São João group seeks to provide integrated solutions for health promotion. The initiative involves collaboration between medical professionals, veterinarians, ecologists, and other experts in managing and controlling threats to public health. The focus is on tracing the spread of diseases among humans, other animals, and the environment. For more information see SNS (2023).
